# Scaled Model Tests Investigating Deformation Characteristics of Geosynthetic Reinforced Soil (GRS) Abutments under Vertical Loads

**DOI:** 10.3390/ma16134601

**Published:** 2023-06-26

**Authors:** Chao Xu, Qingming Wang, Panpan Shen, Geye Li, Qiushen Wang, Xiao Zhang, Chongxi Zhao

**Affiliations:** 1 Department of Geotechnical Engineering, Tongji University, Shanghai 200092, China; 2 Shanghai Investigation, Design & Research Institute Co., Ltd., Shanghai 200434, China

**Keywords:** abutment, deformation, geosynthetics, geosynthetic reinforced soil, volumetric deformation

## Abstract

This study conducted plane-strain scaled model tests to investigate the deformation characteristics of geosynthetic reinforced soil (GRS) abutments subjected to vertical loads. Setback distance, i.e., the distance between the back of the abutment facing and the front of the loading plate, was chosen as the investigated influencing factor since it is one of the most frequently used variables by engineers for the design of GRS abutments. This study analyzed the settlements at the top of the abutment, the lateral displacements of the abutment facing, and the volumetric deformations of the abutment under the applied vertical loads. Test results showed that increasing the setback distance could effectively reduce the deformations of the GRS abutment. There existed an optimum setback distance and further increasing the setback distance beyond this optimum value did not have a significant effect on reducing the abutment deformations. The vertical, lateral, and total volumetric deformations of the GRS abutment showed an approximately linear increase with the increase of the applied vertical loads. The lateral volumetric deformations of the GRS abutment were larger than its vertical volumetric deformations and therefore the total volumetric strains of the GRS abutment were not zero based on the test results. However, the theory of zero volume change may still be suitable for the deformation calculation of the GRS abutment since the values of the volumetric strains were minimal. The measured maximum lateral facing displacements were compared with the calculated values using the US Federal Highway Administration (FHWA) method, which assumes zero volume change of the GRS abutment under vertical loads. Comparison results indicated that the FHWA method overestimated the maximum lateral facing displacements of the GRS abutment under vertical loads. An improved method was proposed in this study to calculate the maximum lateral facing displacements under vertical loads based on the theory of zero volume change and a revised distribution of the settlements at the top of the GRS abutment. Results showed that the improved method could better predict the maximum lateral facing displacements as compared to the FHWA method.

## 1. Introduction

For the last few decades, geosynthetic reinforcement has shown great economic benefits in geotechnical engineering structures and has been widely used in reinforced retaining walls, slopes, and embankments. In recent years, geosynthetic reinforced soil (GRS) with closely spaced geosynthetic layers (i.e., no larger than 0.3 m) has been proven to have the performance of a composite material through the interaction between reinforcement and soil mass, hence the recent emerging use of the GRS as load-carrying structures such as bridge abutments. Different from the traditional reinforced concrete pile-supported abutments illustrated by Fu et al. [1] and Ma et al. [2], GRS abutments have the advantages of reduced construction cost and time [3,4]. In recent years, the US Federal Highway Administration (FHWA) developed a specific bridge system called the Geosynthetic Reinforced Soil–Integrated Bridge System (GRS-IBS). In 2005, Bowman Bridge, constructed in Ohio, USA, was the first application of the GRS-IBS [3]. Since then, more and more GRS-IBSs have been constructed across the USA. Researchers have conducted field monitoring to investigate the long-term performances of the GRS-IBSs under working stress conditions. Results showed that the GRS-IBSs have good service performances and significant advantages in eliminating bumps at the end of the bridge slabs [5,6,7,8,9,10,11,12].

Since the GRS abutment serves as the most important part of the GRS-IBS to directly support the bridge load, its bearing capacity and service performance (e.g., deformation) are extremely important to this specific technology. As an effective and convenient method, numerical analysis has been widely used to investigate the performances of the GRS abutment. Many studies have been published in literature by using finite element or finite difference methods to investigate different influencing factors on the performances of the GRS abutment [13,14,15,16,17,18,19]. Results show that the GRS abutment with close spacing has an excellent service performance and a high load-carrying capacity to meet the design requirements. However, the input parameters for constitutive models in the numerical analysis are based on a large number of laboratory soil tests, which may cause the uncertainty of the numerical results [20]. Raja et al. [21] proposed a novel hybrid artificial intelligence (AI)-based model to predict the load-settlement behavior of the GRS abutment. However, this model was still limited by its training range [21].

Scaled model test is another effective method to simulate engineering prototypes and has been carried out to investigate the performances of the GRS abutment. Mini-pier tests, which treat the reinforced soil mass as a composite material, were constructed to systematically investigate the influences of reinforcement spacing, tensile strength, and backfill properties on the service performances of the reinforced soil mass under vertical loads [22,23,24,25]. Wu et al. [26] evaluated the failure loads of two full-scale segmental-facing GRS abutments and pointed out that the design values were much smaller than the measured loads. In addition, several model tests published in literature [27,28,29] showed that different facing conditions have an influence on the performances of the GRS abutment. However, there are few studies considering the setback distance, i.e., the distance between the back of the abutment facing and the front of the loading plate, as an influencing factor on the performances of the GRS abutment. In the FHWA design guideline [30], the suggested setback distance value for design is based on empirical data and test results. Therefore, it is important for model tests to incorporate the setback distance as an influencing factor.

This study conducted three plane-strain scaled model tests to investigate the effects of the setback distance (*a_b_*), which is the distance between the back of the abutment facing and the front of the loading plate, on the deformation characteristics of the GRS abutment subjected to vertical loads. Since the service performance of the GRS abutment greatly depends on its deformation, this study chose the deformation characteristics rather than the ultimate bearing capacity of the GRS abutment as the investigated subject. Setback distance was chosen as the investigated influencing factor since it is one of the most frequently used variables by engineers for the design of GRS abutments. The analyzed deformation of the GRS abutment included the settlements at the top of the abutment, the lateral displacements of the abutment facing, and the volumetric deformations of the abutment. The FHWA method was selected for the comparison of the calculated maximum lateral facing displacements with the measured data. An improved method was proposed in this study to calculate the maximum lateral facing displacements under vertical loads.

## 2. Model Test Geometry, Material, and Plan

### 2.1. Model Geometry

**Figure 1:**
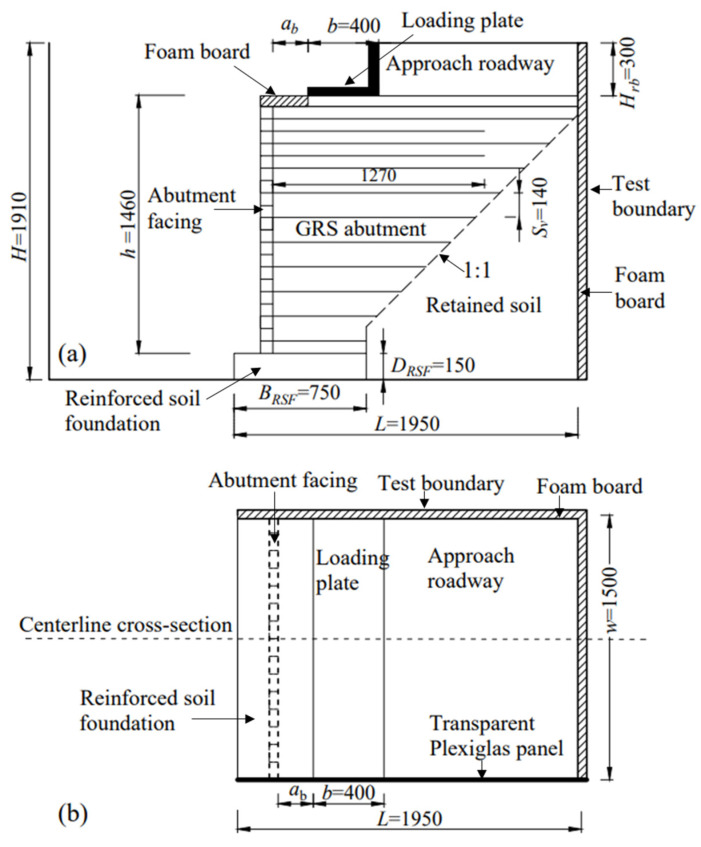
Test configuration of the model GRS abutment: (**a**) Front view and (**b**) top view (unit: mm).

The Bowman Bridge constructed in Ohio, USA [3] with a height of 4.7 m was chosen as the prototype of the model tests conducted in this study. The model abutment was constructed in a test pit with dimensions of 4.8 m (long) × 3.4 m (wide) × 2.0 m (high). Considering the geometry of the test pit and the capacity of the loading device, a length scaling factor of 3 was adopted in this study. It should be noted that using even smaller scaling factors (e.g., 1.5 or 2) in 1-g model tests could better reflect the stress and strain levels of the prototype. However, the limited space of the test pit made it impossible, hence the choice of 3 as the scaling factor. Figure 1 shows the dimensions of the model GRS abutments. The whole model constructed in this study had a total length (*L*) of 1.95 m, a total height (*H*) of 1.91 m, and a total width (*w*) of 1.50 m in the out-of-plane direction. The height of the GRS abutment itself (*h*) was 1.46 m, the width of loading plate (*b*) was 0.40 m, and the cut slope of the retained soil was 1:1. The loading plate was placed on top of the GRS abutment at a setback distance (*a_b_*) from the back of the abutment facing. The lengths of all primary reinforcement layers used in the GRS abutment were such that they reached the cut slope. The reinforcement vertical spacing (*S_v_*) between primary reinforcement layers was 0.14 m. Two layers of bearing bed reinforcement underneath the loading plate were used in the model tests. According to the design guideline provided by the FHWA [30], the length of the bearing bed reinforcement layers should be no smaller than the sum of the width of the loading plate and twice the setback distance (*b* + 2*a_b_*). Therefore, the length of the bearing bed reinforcement was 1.27 m in this study.

### 2.2. Test Material

**Figure 2:**
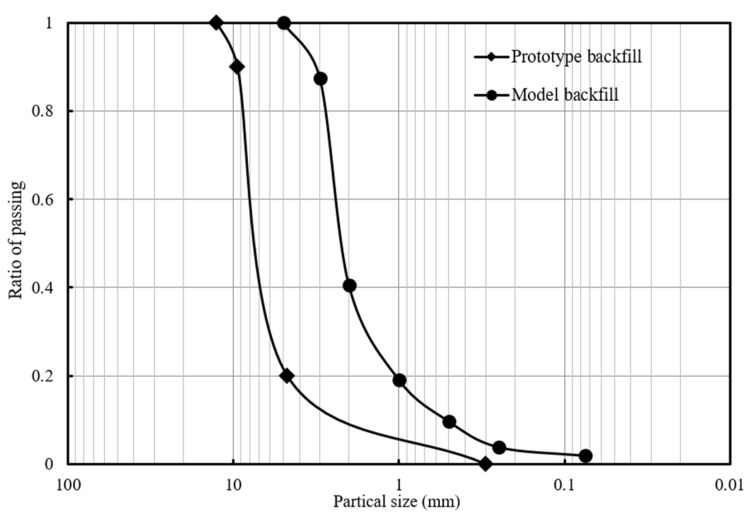
Sieve analysis results of both the prototype and the model backfill.

Open-graded quartz sand was used as backfill in the model tests. Figure 2 shows the gradation curves of both the prototype backfill used in the Bowman Bridge and the model backfill (i.e., open-graded quartz sand). The model backfill used in this study was scale-reduced from the prototype backfill so that the gradation curves of the prototype and the model backfill, as shown in Figure 2, satisfied the similitude relationship. According to the sieve analysis results shown in Figure 2, the particle size of the backfill ranged from 0.1 mm to 4.2 mm. The model backfill had a maximum dry density of 1860 kg/m^3^. During construction, the controlled dry density of the model backfill was 1760 kg/m^3^, which corresponded to a compaction degree of 95%. Triaxial test results showed that the model backfill had a friction angle of 48° and a cohesion of 0 kPa at the compaction degree of 95%.

**Figure 3:**
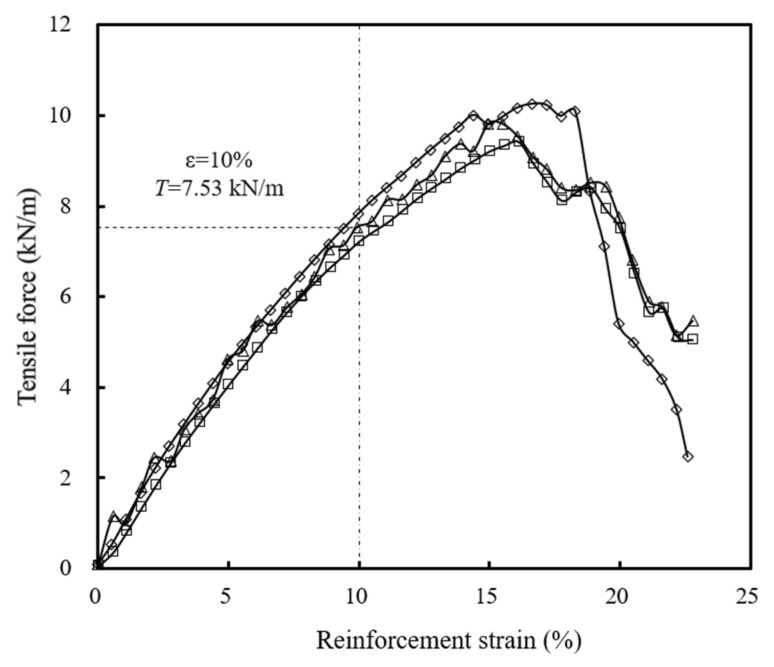
Wide-width tensile tests of model geotextiles used in the tests.

This study selected biaxial woven polypropylene geotextiles as the reinforcement material. Figure 3 shows the results of three wide-width tensile tests conducted on the geotextiles. The average value of the tensile strength at 10% reinforcement strain was 7.53 kN/m for the model geotextile, which corresponded to a tensile strength of 67.77 kN/m for the prototype geotextile at 10% reinforcement strain.

The abutment facing was simulated in the model tests using modular blocks with dimensions of 130 mm (long) × 70 mm (high) × 70 mm (wide). An L-shaped steel loading plate with the dimensions of 1.50 m (long) × 0.40 m (wide) × 0.04 m (thick) was used as the beam seat to simulate the interaction between the bridge girders and the GRS abutment. Concrete blocks were laid behind the loading plate to simulate the approach roadway.

### 2.3. Construction

**Figure 4:**
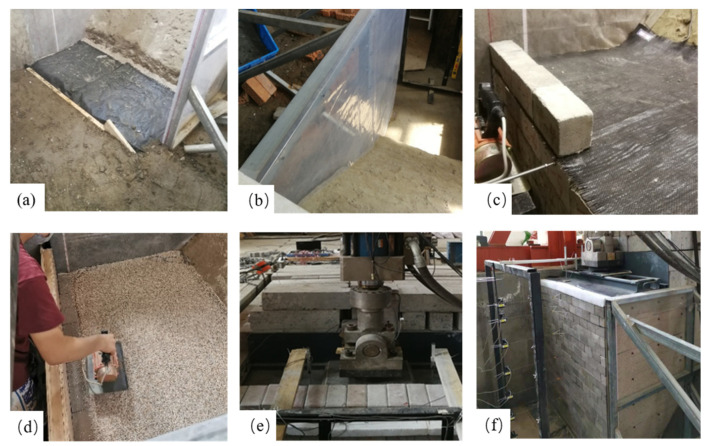
Construction of the model GRS abutment: (**a**) construction of the reinforced soil foundation; (**b**) application of the polytetrafluoroethylene membrane; (**c**) placement of the modular facing blocks; (**d**) backfill compaction; (**e**) placement of the loading device; (**f**) completion of the model GRS abutment.

Before construction, polytetrafluoroethylene membrane and lubricating oil were applied to the inner surface of the test pit and the Plexiglas panel, respectively, to reduce the negative effects of friction on the test results and to ensure the plane-strain condition. The reinforced soil foundation was first constructed using a mass-volume control method, followed by the construction of the GRS abutment in 20 lifts. In each lift, a layer of the modular facing blocks was placed, followed by a layer of the model backfill and the geotextile reinforcement. The top three geotextile layers were connected to the modular facing blocks using double-faced duct tape to simulate the mechanical connection used in the prototype abutment while the remaining geotextile layers were frictionally connected to the modular facing blocks. Finally, the steel loading plate and the concrete blocks simulating the approach roadway were put on top of the GRS abutment. Figure 4 shows the construction process of the model GRS abutment.

### 2.4. Test Plan and Instrumentation

This study investigated the influence of the setback distance (*a_b_*) on deformation characteristics of GRS abutments under vertical loads. Therefore, three tests with different setback distances were conducted in this study, as shown in Table 1.

**Figure 5:**
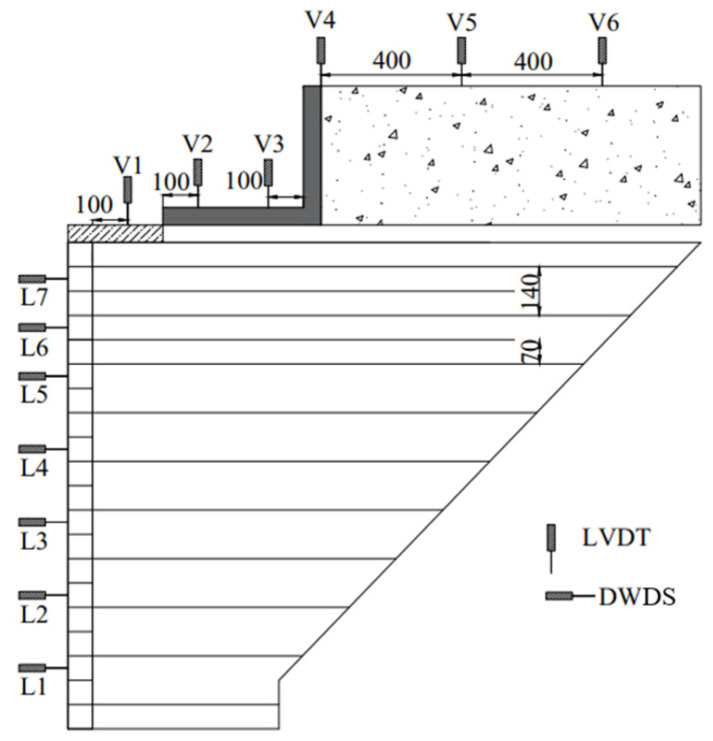
Instrumentation layout (Unit: mm).

Figure 5 shows the instrumentation layout when *a_b_* was 0.2 m. The instrumentation layouts for the other two *a_b_* were similar and omitted to save space. Six Linear Variable Differential Transformers (LVDTs) were installed at the top of the GRS abutment to monitor the settlements. Among these six LVDTs, only 2 (i.e., V2 and V3) were used to monitor the settlements of the loading plate. The loading plate is a rigid plate and steel ribs were used to reinforce the loading plate to ensure its rigidity. Therefore, it was assumed that no bending deformation of the loading plated occurred under vertical loading and the settlements of the loading plate were linearly distributed along its width. Using two LVDTs was considered sufficient to capture the linear distribution of the settlements. In addition, the limited area at the top surface of the loading plate resulted in difficulties in placing additional LVDTs since the loading device occupied most of the space. Lateral displacements of the abutment facing were not uniformly or linearly distributed along the abutment height due to the flexibility of the facing consisting of multiple modular blocks. Discretization of the lateral facing displacements was considered in this study by using seven draw-wire displacement sensors (DWDS) installed in front of the abutment facing to monitor the lateral displacements.

It should be noted that all the sensors were installed at the centerline cross section along the out-of-plane direction of the GRS abutment, as shown in Figure 1b. The readings of all the sensors were zeroed out after the completion of construction prior to loading. In other words, the results of all these sensors represented the deformations of the GRS abutment induced by vertical loading only.

For each test, vertical loads were applied to the GRS abutment by multi-stage loading with increments of 10 kN. According to the method recommended by the FHWA design guideline [30], the ultimate bearing capacity of the model GRS abutment was 30 kN. Considering this calculated ultimate bearing capacity as well as the capacity of the loading device, this study adopted 90 kN as the maximum applied load, which was three times the calculated ultimate bearing capacity and should fully cover the range of the working stress condition of the GRS abutment. In other words, this 90 kN maximum applied load fully satisfied the purpose of this study, which was to investigate the deformation characteristics of the GRS abutment subjected to vertical loads. The stage loading was terminated when one of the following conditions occurred: (1) The global failure of the GRS abutment occurred, (2) the settlement of the top of the abutment reached 5% of the abutment height, or (3) the maximum lateral displacement of the abutment facing reached 10% of the abutment height.

## 3. Test Results

The model GRS abutment did not show significant deformation when the applied loads reached the predetermined maximum load of 90 kN. In order to obtain greater deformation values and better investigate the deformation characteristics of the GRS abutment, stage loading was not terminated and the applied load continued increasing to 100 kN, which was the maximum capacity of the loading device. No obvious failure phenomenon was observed in any of the three model tests, indicating that all the three model GRS abutments were under working stress conditions.

### 3.1. Settlement at the Top of the Abutment

**Figure 6:**
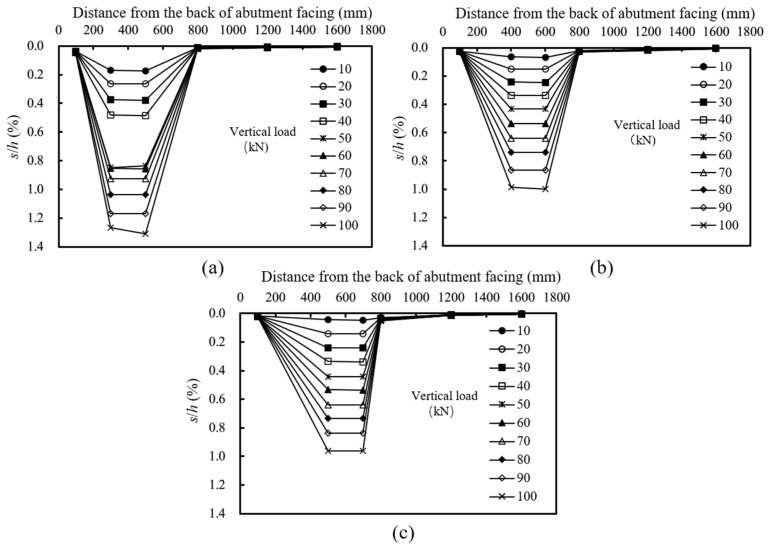
Distributions of normalized settlements at the top of the GRS abutment under loading: (**a**) T1: *a_b_* = 0.20 m; (**b**) T2: *a_b_* = 0.30 m; (**c**) T3: *a_b_* = 0.40 m.

Figure 6 shows the distributions of the normalized settlements at the top of the abutment subjected to different applied loads. The normalized settlement was calculated as the ratio of the settlement *s* to the abutment height *h*.

Figure 6 indicates that the change of the setback distance did not produce significant effects on the distributions of the normalized settlements at the top of the GRS abutment. The settlements under the loading plate were obviously larger than those in other areas and the settlements increased approximately linearly with the increase of the applied vertical load in all tests, indicating that the model abutments were still under working stress conditions. In T1, when the applied load increased from 40 kN to 50 kN, the settlement increased significantly. However, the increment of the settlement decreased in the subsequent loading stages. It was speculated that a malfunction of the loading device occurred at this loading stage, thus resulting in the significant increase of the normalized settlement. Figure 6 also shows that with the increase of the setback distance, the settlement under the loading plate decreased in the beginning and then remained approximately stable, indicating that increasing the setback distance could reduce the settlement at the top of the abutment, but there existed an optimum setback distance (*ab*)*_opt_* and further increasing the setback distance beyond this optimum value did not have a significant effect on reducing the abutment settlements.

### 3.2. Lateral Facing Displacement

**Figure 7:**
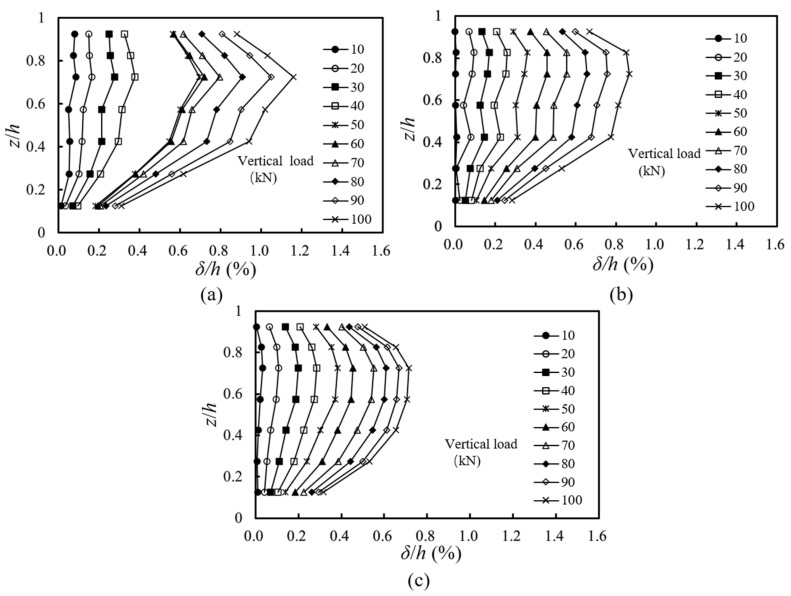
Distributions of normalized lateral facing displacements along the abutment height under loading: (**a**) T1: *a_b_* = 0.20 m; (**b**) T2: *a_b_* = 0.30 m; (**c**) T3: *a_b_* = 0.40 m.

Figure 7 shows the distributions of normalized lateral facing displacements along the abutment height under different applied loads. The normalized lateral facing displacement was calculated as the ratio of the lateral facing displacement *δ* to the abutment height *h*. As expected, with the increase of the applied loads, the lateral facing displacements increased. The maximum and minimum lateral facing displacements occurred near 1/3 *h* from the top of the abutment and near the bottom of the abutment, respectively. Comparing T1, T2, and T3, it could be found that with the increase of the setback distance, the location of the maximum lateral facing displacement gradually moved from the top to the mid-height of the abutment.

In addition, as compared with the other two cases, T1 with the smallest setback distance had a maximum lateral facing displacement of approximately 1.2%*h* under the applied load of 100 kN, which did not exceed the allowable lateral facing displacement of 2%*h* under working stress conditions according to the design guideline published by the FHWA [30]. In T1 with the smallest setback distance, when the vertical load was larger than 50 kN, the increments of the lateral facing displacements increased gradually with the increase of the applied load. In T2 when the setback distance increased to 0.30 m, the increments of the lateral facing displacements under each loading stage were approximately the same. In T3 when the setback distance further increased to 0.40 m, the increments of the lateral facing displacements decreased with the increase of the applied load.

Adams et al. [31] proposed a zero volume change assumption based on the mini-pier tests. This assumption was adopted by the FHWA design guidelines [30] to calculate the maximum lateral displacement of the GRS abutment. This assumption indicates that the volume lost at the top of abutment due to compression is equal to the volume gained at the facing due to lateral deformation under vertical loads. Few studies were published in literature analyzing the volumetric deformation of GRS abutments. Therefore, further research is needed to analyze the volumetric deformations of the GRS abutment under vertical loads.

### 3.3. Volumetric Deformation

**Figure 8:**
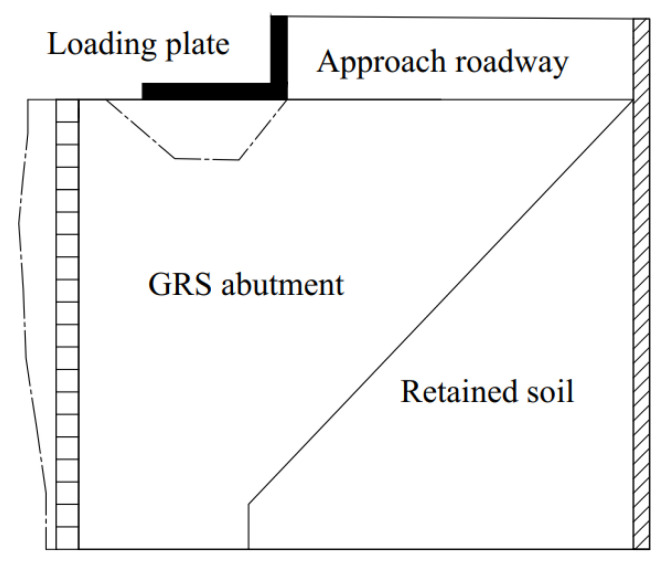
Schematic of the deformed GRS abutment based on the monitored results.

Figure 8 shows the schematic of the deformed GRS abutment based on the measured lateral facing displacements as well as the top settlements. Under the applied vertical loads, the top of the GRS abutment settled and the abutment facing expanded laterally. The vertical volumetric deformation of the GRS abutment due to the applied load can be calculated as Δ*V_v_* = −Δ*S_v_* × *w*, where *w* is the width of the GRS abutment in the out-of-plane direction (*w* = 1.5 m in this study) and Δ*S_v_* is calculated by integrating the settlement at the top of the GRS abutment along its length direction. According to the settlement distributions of the GRS abutment as shown in Figure 6, the reading of LVDTs V1, V4, V5, and V6 were minimal under different applied loads. In other words, the settlement of the abutment occurred mainly underneath the L-shaped loading plate. Therefore, the calculation of the vertical volumetric deformation of the GRS abutment did not consider the settlements behind the loading plate (i.e., the area of the approach roadway) and the settlements close to the abutment facing (i.e., the area in front of the LVDT V1). The negative sign in front of Δ*S_v_* indicated that the vertical compression was deemed as negative during the volumetric deformation calculation. The lateral volumetric deformation of the GRS abutment due to the applied load can be calculated as Δ*V_l_* = Δ*S_l_* × *w*, where Δ*S_l_* is calculated by integrating the lateral displacements of the abutment facing over the abutment height. During the calculation of Δ*S_l_*, this study assumed that the lateral displacement at the top and the bottom of the abutment facing were the same as those measured by the DWDSs L7 and L1 (as shown in Figure 5), respectively.

The vertical volumetric strain of the GRS abutment was defined as Δ*V_v_*/*V*_0_, where *V*_0_ was the original volume of the GRS abutment (*V_0_* = 2.40 m^3^ in this study). Similarly, the lateral volumetric strain of the GRS abutment was defined as Δ*V_l_*/*V*_0_. Therefore, the total volumetric strain of the GRS abutment was *ε_v_* = (Δ*V_v_* + Δ*V_l_*)/*V*_0_.

**Figure 9:**
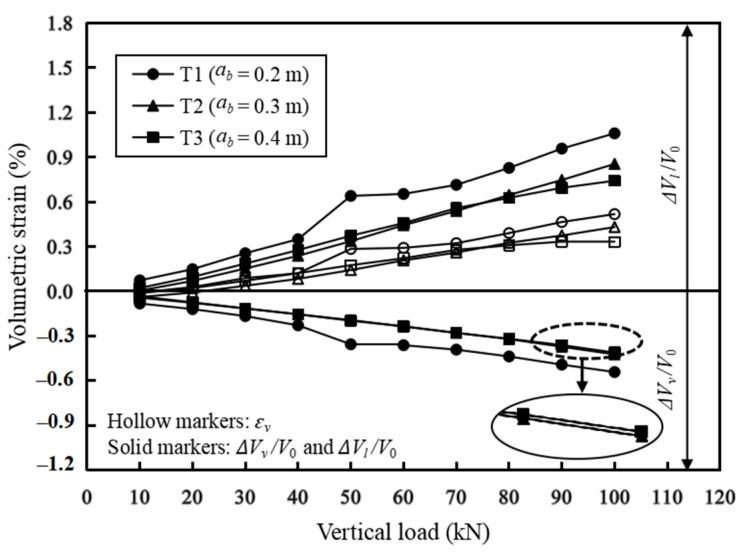
Effects of the setback distance on the volumetric deformations of the GRS abutment under vertical loads.

Figure 9 shows the effects of the setback distance on the volumetric strains of the GRS abutment under different vertical loads. The vertical, lateral, and total volumetric strains of the GRS abutment showed approximately linear increases with the increase of the applied vertical loads. Meanwhile, Figure 9 shows that the setback distance did have some influences on the volumetric deformation of the GRS abutment. The maximum volumetric strain of the GRS abutment was 0.52% under the 100 kN vertical load in T1. In T2 and T3, the volumetric strains of the GRS abutment were similar and both of them were smaller than that in T1. In other words, increasing the setback distance could reduce the vertical and lateral volumetric deformations of the GRS abutment. However, there existed an optimum setback distance (*a_b_*)*_opt_* and further increasing the setback distance beyond this optimum value did not have a significant effect on reducing the volumetric deformations of the GRS abutment.

In addition, Figure 9 also shows that, despite the change of the setback distance, the lateral volumetric deformations of the GRS abutment were always larger than its vertical volumetric deformations and the differences between the lateral and vertical volumetric deformations increased with the increase of the vertical load. Therefore, the total volumetric strain of the GRS abutment was not zero based on the test results. It should be pointed out that the theory of zero volume change was based on the results of mini-pier tests. The axisymmetric boundary condition of the mini-pier tests was significantly different from the two-dimensional plane-strain boundary condition of the GRS abutment. The differences of the boundary conditions between mini-piers and abutments could be the reason causing the non-zero volumetric deformations of the GRS abutments in this study. However, Figure 9 also shows that the total volumetric strain of the GRS abutment was less than 0.5%. Therefore, the theory of zero volume change may still be suitable for the deformation calculation of GRS abutments.

### 3.4. Comparison with FHWA Method

**Figure 10:**
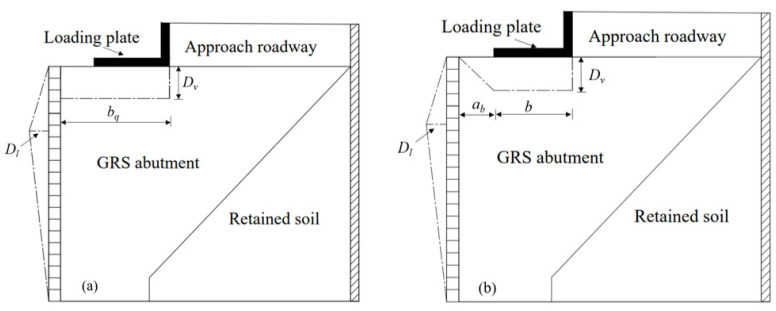
Assumed distributions of the volumetric deformation of the GRS abutment under vertical loading: (**a**) The FHWA method; (**b**) the improved method proposed in this study.

**Figure 11:**
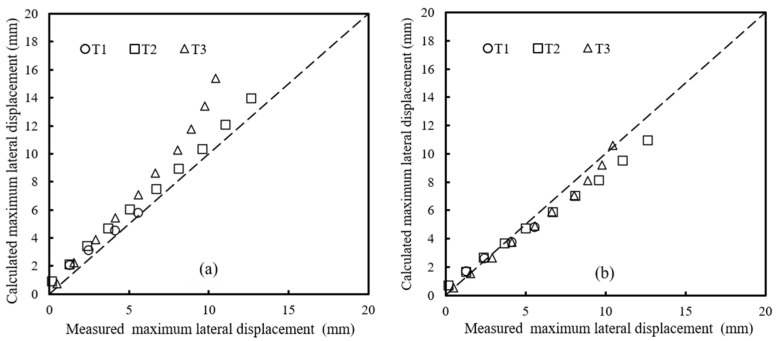
Comparison between the calculated and the measured maximum lateral facing displacements of the abutment facing under vertical loading: (**a**) calculated using the FHWA method; (**b**) calculated using the improved method proposed in this study.

The FHWA [30] proposed the following Equation (1) to predict the maximum lateral facing displacement (*D_L_*) of the abutment based on the theory of zero volume change. This equation also assumed a triangular distribution of the lateral facing displacements along the abutment height and a uniform distribution of the settlements at the top of the abutment, as shown in Figure 10a:

(1) $$D_{L}=\frac{2b_{q}D_{v}}{h}=\frac{2\left(a_{b}+b\right)D_{v}}{h}$$

where *b_q_* is the width of the load area along the top of the abutment including the setback distance (i.e., *b_q_* = *a_b_* + *b*), *D_v_* is the vertical settlement at the top of the GRS abutment, and *h* is the height of the GRS abutment. Figure 11a shows the measured lateral facing displacements from the tests and the calculated values using Equation (1) proposed by the FHWA. Due to the malfunction of the loading device which occurred in T1 when the applied load increased from 40 to 50 kN, the measured results of T1 under the applied load larger than 40 kN were not presented in Figure 11a. Figure 11a indicates that the FHWA method overestimated the lateral facing displacements under vertical loads under all three different setback distances, which was contrary to the results from filed monitoring by Saghebfar [6]. The model tests conducted in this study were under two-dimensional plane-strain condition, which was different from the three-dimensional GRS abutment conducted in the field [6]. Different boundary conditions between the model and the field GRS abutments may result in the different volumetric deformation results.

In addition, Figure 11a also shows that, with the increase of the setback distance, the deviations of the calculated results from the measured ones became larger, indicating that the setback distance had a significant influence on the maximum lateral facing displacement. Test results in Figure 6 show that the settlements under the setback distance area were obviously different from those under the beam seat width, indicating that it is not reasonable for the FHWA method to assume a uniform distribution of the settlements at the top of the GRS abutment under vertical loads. In other words, it was speculated that FHWA’s assumption of uniformly distributed settlements at the top of the GRS abutment could be the reason for overestimating the maximum lateral facing displacements under vertical loads.

A revision was made based on the test results in this study to the uniform distribution of the settlements at the top of the GRS abutment adopted by the FHWA. As shown in Figure 10b, the distributions of the settlements under the setback distance area and the beam seat width were assumed to be triangular and uniform, respectively. The triangular distribution of the lateral facing displacements along the abutment height was kept the same as that in the FHWA method. Under the theory of zero volume change and the revised settlement distribution, an improved method was proposed in this study to calculate the maximum lateral facing displacement of the GRS abutment under vertical loads, shown as the following Equation (2):

(2) $$D_{L}=\frac{\left(a_{b}+2b\right)D_{v}}{h}$$

Figure 11 shows the comparison between the FHWA method (i.e., Equation (1)) and the improved method proposed in this study (i.e., Equation (2)). The comparison between Figure 11a,b shows that the improved method could better predict the maximum lateral facing displacements as compared to the FHWA method. However, other parameters such as the beam seat width and the abutment height could also have influences on the calculation of the maximum lateral facing displacements. Further research is needed to investigate the distributions of the volumetric deformations of the GRS abutment under vertical loads.

## 4. Limitations

It is important to acknowledge the limitations of this study, which could be helpful for the improvements of future research. Due to the fact that constructing and loading the model GRS abutments were time- and labor-consuming as well as technique-challenging, only three model tests were conducted in this study investigating three different setback distances. The conclusions of this study were limited by the number of the model tests. The boundary conditions of the model tests conducted in this study (i.e., two-dimensional plane-strain conditions) are different from those in the field (i.e., three-dimensional conditions). The two-dimensional boundary condition generally results in more conservative results (e.g., larger deformations under the same vertical loads) as compared to the three-dimensional boundary condition. Although test results of this study indicated that the volumetric deformation of the GRS abutment under vertical loads was not zero, the zero volume change assumption made by the FHWA may still be suitable for the design of GRS abutments since the value of the volumetric strains were less than 0.5%. Therefore, both the FHWA method and the improved method proposed in this study were based on the zero volume change assumption. The improved method revised the distribution of the settlements at the top of the GRS abutment. Further studies may be necessary to investigate the volumetric distributions of the GRS abutment under vertical loads in order to give better predictions of the deformations. In addition, the humidity of the backfill was not considered in this study. Despite the above limitations, the results of this study provided valuable insights into the influence of the setback distance on the deformation characteristics of the GRS abutments under working stress conditions and gave important references for engineering applications.

## 5. Conclusions

In this study, two-dimensional plane-strain scaled model tests were conducted to investigate the deformation characteristics of geosynthetic reinforced soil (GRS) abutments subjected to vertical loads. Setback distance, which is the distance between the back of the abutment facing and the front of the loading plate, was chosen as the investigated influencing factor since it is one of the most frequently used variables by engineers for the design of GRS abutments. This study analyzed the settlements at the top of the abutment, the lateral displacements of the abutment facing, and the volumetric deformations of the GRS abutment under the applied vertical loads. The US Federal Highway Administration (FHWA) method was selected for the comparison of the calculated maximum lateral facing displacements with the measured data. An improved method was proposed in this study to calculate the maximum lateral displacements of the abutment facing under vertical loads. The following conclusions can be made from this study:

(1) Increasing the setback distance could effectively reduce the settlements at the top of the abutment, the lateral facing displacements, and the volumetric deformations of the GRS abutment. However, there existed an optimum setback distance (*a_b_*)_opt_ and further increasing the setback distance beyond this optimum value did not have a significant effect on reducing the abutment deformations.
(2) With the increase of the applied vertical loads, the vertical, lateral, and total volumetric deformations of the GRS abutment increased linearly. The lateral volumetric deformations of the GRS abutment were larger than its vertical volumetric deformations and therefore the total volumetric strains of the GRS abutment were not zero based on the test results. However, the theory of zero volume change may still be suitable for the deformation calculation of GRS abutments since the values of the volumetric strains were minimal.
(3) A comparison between the measured maximum lateral facing displacements and the calculated values using the method proposed by the FHWA showed that the FHWA method overestimated the lateral facing displacements under vertical loads. It was speculated that the FHWA’s assumption of uniformly distributed settlements at the top of the GRS abutment could be the reason for overestimating the maximum lateral displacements.
(4) An improved method was proposed in this study to calculate the maximum lateral facing displacements under vertical loads based on the theory of zero volume change and a revised distribution of the settlements at the top of the GRS abutment. Results showed that the improved method could better predict the maximum lateral facing displacements as compared to the FHWA method.
(5) Results of this study could provide valuable insights into the influence of the setback distance on the deformation characteristics of the GRS abutment under working stress conditions and give important references for engineering applications of GRS abutments. A reasonable setback distance has the advantages of both reducing the bridge span and controlling the abutment deformations.

## Figures and Tables

**Table 1:** Model test plan.

No.	Setback Distance *a_b_* (m)	Reinforcement Spacing *S_v_* (m)
T1	0.2	0.14
T2	0.3	0.14
T3	0.4	0.14

Note: *a_b_* represented the setback distance, i.e., the distance between the back of the abutment facing and the front of the loading plate.

## Data Availability

Not applicable.
